# Effect of a community-based intervention for cardiovascular risk factor control on stroke mortality in rural Gadchiroli, India: study protocol for a cluster randomised controlled trial

**DOI:** 10.1186/s13063-019-3870-x

**Published:** 2019-12-23

**Authors:** Yogeshwar Kalkonde, Mahesh Deshmukh, Sindhu Nila, Sunil Jadhao, Abhay Bang

**Affiliations:** Society for Education, Action and Research in Community Health (SEARCH), Shodhgram, Post-Chatgaon, Taluka-Dhanora, District-Gadchiroli, Maharashtra 442605 India

**Keywords:** Stroke, Prevention, Mortality, Hypertension, Diabetes, Treatment, Trial, Rural, India, Community health worker

## Abstract

**Background:**

Stroke has emerged as a leading cause of death in rural India. However, well-tested healthcare interventions to reduce stroke mortality in rural under-resourced settings are lacking. The aim of this study is to evaluate the effect of a community-based preventive intervention on stroke mortality in rural Gadchiroli, India.

**Methods:**

The study is a two-arm, parallel group, cluster randomised controlled trial in which 32 villages will be randomised to the intervention and the enhanced usual care (EUC) arm. In the intervention arm, individuals ≥50 years of age will be screened for hypertension, diabetes and stroke by trained Community Health Workers (CHWs). Screened individuals who are positive will be referred to a mobile outreach clinic which will visit the intervention villages periodically. A physician in the clinic will confirm the diagnosis, provide guideline-based treatment and follow up patients. The CHWs will make home visits once a month to ensure medication compliance and counsel patients to reduce salt consumption and quit tobacco and alcohol. In the EUC arm, households will be provided information on the ill effects of tobacco use and steps to quit it. Individuals from both the arms will have access to the government’s national programme for the prevention and control of non-communicable diseases, where treatment for hypertension, diabetes and preventive treatment after stroke is available at the nearest primary health centres (PHCs). The intervention will be implemented for 3.5 years. The primary outcome will be a reduction in stroke mortality in the last 2.5 years of the intervention.

**Discussion:**

This trial will provide important information regarding the feasibility and effect of a community-based preventive intervention package on stroke mortality in a rural under-resourced setting and can inform India’s non-communicable diseases prevention and control programme. If successful, such an intervention can be scaled up in the rural regions of India and other countries.

**Trial registration:**

Clinical Trials Registry of India: CTRI/2015/12/006424. Registered on 8 December 2015.

## Background

Epidemiological transition from communicable to non-communicable diseases has occurred in India [1–3]. Nearly two thirds of India’s population lives in rural areas [4] and studies conducted by us and others have shown that stroke has emerged as a major public health problem in these areas [1, 5, 6]. In rural Gadchiroli in India, stroke was the leading cause of death and accounted for 14% of all deaths [5]. The prevalence of stroke was also high (535/100,000 population) [6]. Stroke is also a global health priority and is a leading cause of death in the world [7, 8]. The burden of disease due to stroke has now shifted from higher income countries to low- and middle-income countries (LMICs), with 71% of stroke deaths occurring in these countries [9]. Large populations in these LMICs live in rural areas and do not have easy access to preventive or curative care for stroke. The consequences of stroke could be severe in such communities, where resources are scarce. Therefore, well-tested interventions to reduce stroke deaths in the rural areas of India and other LMICs are urgently needed. However, such interventions are currently lacking.

Stroke is preventable. The INTERSTROKE study has identified ten modifiable risk factors, which account for 90% of the risk of stroke [10]. Among these, hypertension is the leading risk factor and accounts for approximately 50% of the risk of stroke [10]. Hypertension control, therefore, emerges as an important target for stroke prevention. Hypertension is also the leading risk factor for death, killing 9 million people globally every year [11], and the World Health Organization considers hypertension control a ‘best buy’ to reduce cardiovascular deaths [12]. Despite this, the screening, diagnosis and control of hypertension remains poor in rural India. In a nationally representative study, hypertension was undetected in 60% of women and 70% of men in rural India, and it was controlled in only 50% of those who were diagnosed with hypertension [13]. Thus, only 15–20% of patients with hypertension had their blood pressure adequately controlled. Another large study based on the fourth round of the National Family Health Survey (NFHS-4) showed similar findings [14]. Among rural individuals aged 15–49 years diagnosed with hypertension, only 72% had received a previous blood pressure measurement, 42% were aware of their diagnosis, 12% were treated, and only 8% had achieved control [14].

Diabetes is another risk factor for stroke, and the control of diabetes also remains poor in rural India. A large multi-state study conducted by the Indian Council of Medical Research and involving 14,277 participants showed that good glycaemic control (HbA1c < 7%) was present in only 30% of individuals with diabetes [15]. Secondary prevention of stroke also remains poor in India [16]. The Prospective Urban Rural Epidemiological (PURE) study, which included patients from rural India, found that only 27% of patients in the rural regions of LMICs were receiving anti-platelet agents [17]. Collectively, these studies highlight a significant care gap in stroke prevention in rural India, where 12% of the world’s population lives.

Several challenges exist to the prevention and control of stroke and its risk factors in rural India. Awareness about these conditions remains low [18–23]. Hypertension and diabetes often do not have symptoms, and their diagnosis gets delayed. Screening facilities are not widely available, and even after screening, only a fraction of patients receive a confirmed diagnosis. Continuity of care remains a challenge because of the lack of affordability, accessibility and prioritization of care, leading to suboptimal control of these conditions. To address the rising burden of non-communicable diseases (NCDs), the Government of India has launched an ambitious National Programme for Prevention and Control of Cancer, Diabetes, Cardiovascular diseases and Stroke (NPCDCS). This programme has started screening individuals at the village level, and for diagnosis and treatment, individuals have to travel to the Primary Health Centres (PHCs), the closest point of contact with a physician in the public health care system in rural areas, where treatment is provided free of cost [24]. However, implementation of the programme has been slow and travel to the PHCs is challenging because of a lack of transportation facilities in rural areas.

To reduce stroke mortality, we have designed a community-based healthcare delivery intervention package which tries to address major barriers to the effective control of hypertension and diabetes and to the secondary prevention of stroke in a rural under-resourced setting in India.

## Methods

### Study hypothesis

The study will test the hypothesis that a community-based healthcare delivery intervention for primary and secondary prevention of stroke is feasible and will reduce stroke mortality in a rural community in Gadchiroli.

### Objectives

1. The first objective of the study is to identify the effect of the trial intervention on the following:
  - stroke mortality
  - all-cause and cardiovascular mortality
  - percentage of hypertensive patients taking blood pressure medications in the community
  - blood pressure control
  - blood glucose control
  - awareness about stroke and its risk factors
2. The second objective is to identify the facilitators and barriers to the delivery of the intervention.
3. The final objective is to document the intervention delivery and costs of the intervention to provide insights for scale-up if the intervention is found to be successful.

### Trial design

The study is a community-based, two-arm, parallel-group, cluster randomised controlled trial. As the intervention package is community-based, a cluster randomised design was preferred over individual randomisation.

### Study site and population

**Fig. 1:**
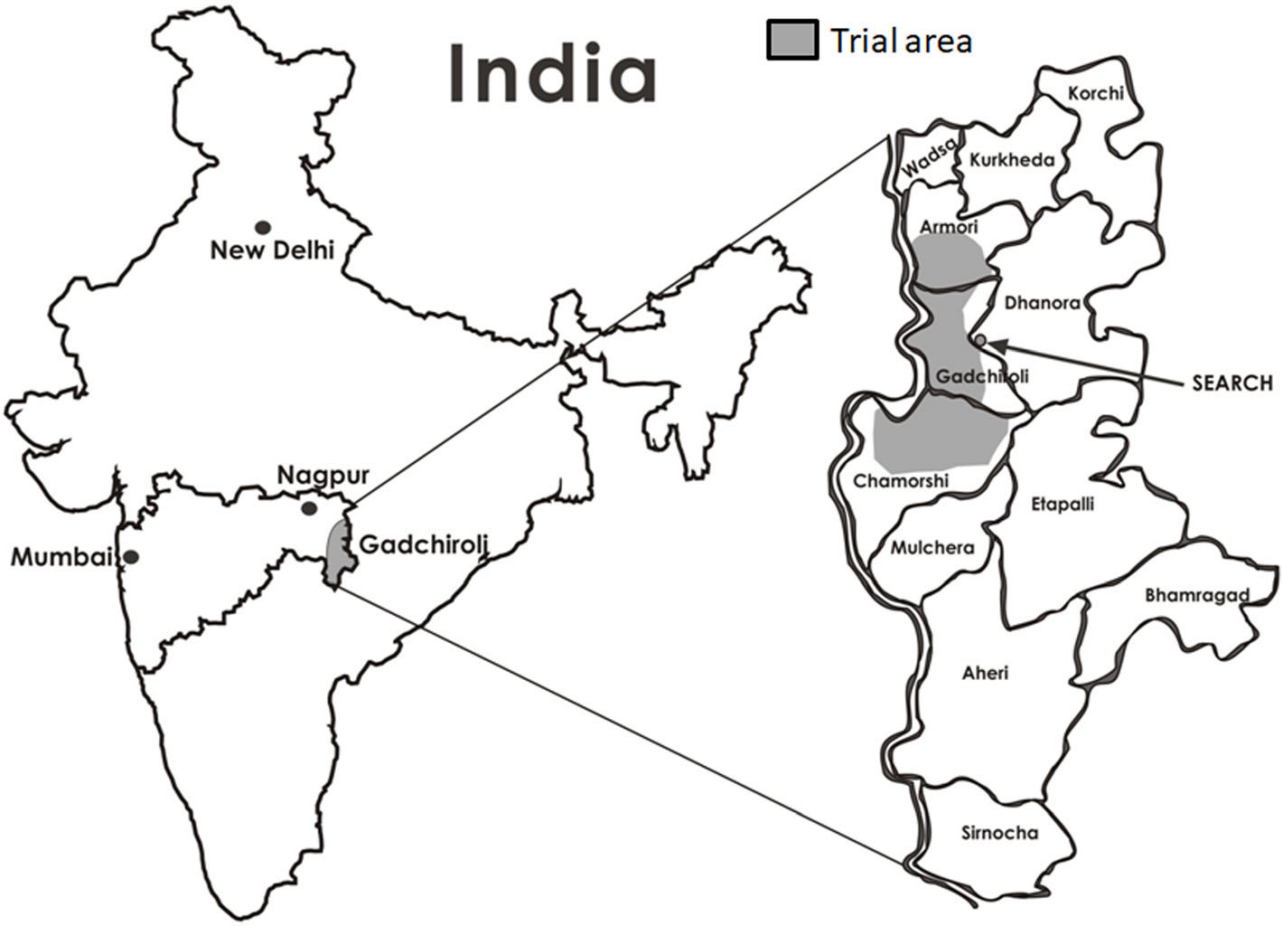
Map of the trial site in Gadchiroli, India

The study will be conducted in Gadchiroli, one of the most underdeveloped districts of India [25]. Gadchiroli is located in the state of Maharashtra in central India (Fig. 1). According to the Indian National Census 2011, the total population of the district was 10,72,942 [26]. Ninety percent of the population of this district lives in rural areas, the literacy rate is 66%, and farming and manual labour are the predominant occupations in the district [26].

The government healthcare services remain an important source of healthcare in the district and provide care through one district hospital (DH), 12 rural or sub-district hospitals (SDH), 45 primary health centres (PHCs) and 365 sub-centres [27]. Each SDH typically covers a population of 100,000, whereas one PHC covers a population of 20,000–30,000. Care for hypertension, diabetes and preventive care for stroke is available free of cost at the PHCs, SDHs and the DH through the NPCDCS. Community level workers referred to as ASHAs have recently started screening of individuals with hypertension, diabetes and cancer at the village level through this programme. In addition to the government-run health services, care is also provided by formal and informal private healthcare providers and some non-governmental organisations. After acute stroke, patients are either admitted to the district hospital, private hospitals or seek care from herbal medicine providers. Brain imaging is not easily available because only one computerized tomography (CT) scanner is available in the entire district. In a population-based study conducted by us in the villages of Gadchiroli, only 12% of stroke patients received brain imaging [6]. Facilities to give intravenous tissue-plasminogen activator (t-PA) also are not available in the district.

SEARCH (Society for Education, Action and Research in Community Health) is a non-governmental organization working in the Gadchiroli district since 1986. It has a service area of 86 villages distributed in three administrative blocks (Gadchiroli, Armori and Chamorshi, Fig. 1) of the district. In these villages, SEARCH has an active demographic surveillance system where all births and deaths are regularly recorded. Information on the cause of death is obtained on all deaths through verbal autopsies [28]. A population census is conducted every 10 years, and the last census was conducted in 2015. A population register is maintained, which is updated annually. SEARCH also runs a rural hospital where primary and secondary level outpatient and inpatient care is provided at a nominal cost and includes care for hypertension, diabetes and stroke. SEARCH, in collaboration with the state government of Maharashtra, also runs a district-wide campaign to reduce the consumption of tobacco and alcohol by generating awareness, enforcing legal restrictions on sales and mobilising communities.

### Randomisation and blinding

**Fig. 2:**
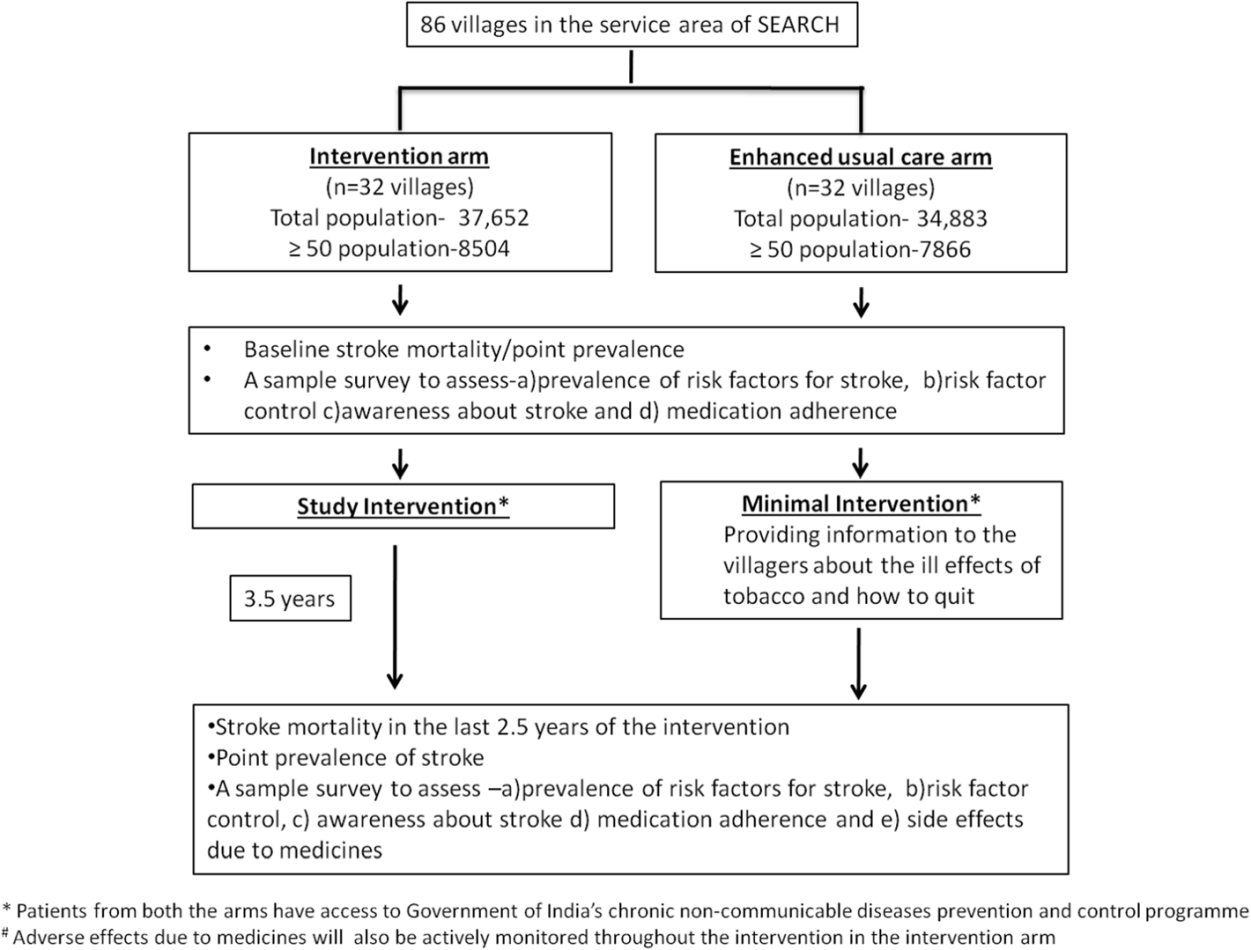
Trial scheme

From the sampling frame of 86 villages in the service area of SEARCH, 32 villages will be randomly assigned to the intervention arm and 32 to the EUC arm (Fig. 2) so that an equal number of villages from each of the three blocks are assigned per arm. Allocation of the villages will be done by the statistician at SEARCH using the random number generator in the statistical software Stata (College Station, TX, USA). Randomisation will be done before the participants are recruited.

Due to the nature of the intervention, blinding the participants or the intervention implementers to the allocation group is not possible. However, the primary outcome assessors will be blinded to the allocation group.

### Inclusion and exclusion criteria

Villages will be included in the study if a) they belonged to the service area of SEARCH and b) have a population >400 so that the average village size of the selected villages is close to the average village population in India, which is approximately 1250 [29]. The largest population for a village in the service area is 2372. Villages within 5 km of Gadchiroli town will be excluded because the purpose of the study is to assess the impact of the intervention in the larger rural communities that have difficulty accessing healthcare.

Individuals from the selected villages will be recruited if they have been residents of the village for >6 months and are ≥50 years of age at the time of screening. We selected this age group for the intervention as >90% of stroke deaths and prevalent strokes in rural Gadchiroli occurred in this age group [5, 6]. To receive the treatment intervention, the individual should have one of the following: a) hypertension, defined as systolic blood pressure ≥140 mmHg and/or diastolic blood pressure ≥90 mmHg or should be on antihypertensive medications at the time of screening by the CHWs which is further confirmed in the outreach clinic by a physician; b) diabetes, defined as glucosuria during screening by the CHWs and random capillary blood glucose of ≥200 mg/dL in the outreach clinic, outside records showing fasting plasma glucose ≥126 mg/dL, a single random venous blood glucose value of ≥200 mg/dL, or previous diagnosis of diabetes and taking treatment for it; or c) has had a stroke, defined using the World Health Organization’s (WHO) definition of stroke as a focal (or at times global) neurological impairment of sudden onset, lasting >24 h (or leading to death) and of presumed vascular origin [30]. Patients diagnosed with hypertension, diabetes or stroke will be invited to receive the trial intervention if they agree to provide a written or audio consent (for individuals who cannot read or write). Those who are terminally ill will be excluded, and those who have symptoms of acute cardiovascular compromise (suspected acute cardiac chest pain; heart failure; or cardiac arrhythmia with pulmonary oedema, hypotension or hypoxia) or kidney diseases (oliguria, clinically suspected acidosis, or pulmonary oedema) that cannot be managed in a primary care setting at the time of evaluation by the outreach physician will be referred for higher care and will be excluded until their clinical condition is unstable.

### Recruitment

Individuals will be recruited to the trial from the villages selected in the intervention arm. A list of individuals ≥50 years of age at the time of commencement of the trial will be drawn from the census conducted by SEARCH in 2015. These individuals will be contacted by the trained CHWs after making a home visit and offered screening followed by treatment for hypertension, diabetes and secondary prevention of stroke.

### Intervention

The intervention package was designed while considering the risk factors for stroke [10], local relevance of these risk factors, current barriers to treatment (Table 1), feasibility of delivering the intervention and preferences of the community regarding providers and their roles. The intervention will be community based, and all the services will be delivered at the level of the community [31–33]. Hypertension, diabetes, alcohol and tobacco use will be targeted. Obesity and lack of physical activity will not be targeted because these are not locally important risk factors given that the population is lean and is engaged in physical labour. Modifications of diet and addressing psychosocial factors were not considered feasible given limited food choices and the lack of an available effective intervention to target psychosocial factors in this area. In a formative study conducted before starting the trial, people felt that it would be convenient for them if a physician could visit the village and then a village-level worker could follow up patients and provide counselling.

**Table 1:** Common barriers to effective control of hypertension, diabetes and secondary prevention of stroke and interventions to address these barriers

Barriers to effective control	Intervention
Lack of awareness about diseases and their risk factors	• Mass awareness programme for hypertension, diabetes and stroke • Individual patient education using culturally appropriate awareness material • Counselling about risk factors (e.g. tobacco, alcohol and high salt use)
Lack of screening facilities at the village level	• Home-based screening of individuals ≥50 years of age for hypertension, diabetes and stroke
Lack of confirmation of diagnosis	• Referral of screen positive individuals to the village outreach clinic • Evaluation and confirmation of diagnosis of hypertension, diabetes and stroke by the OP
Difficulty accessing healthcare facilities on regular basis	• Periodic village clinic by the OP • Monthly home visits by the CHW to reduce need to travel outside the village for seeking care
Lack of treatment adherence	• Monthly follow-up by the CHW by making a home visit • Follow-up by the OP every 2–3 months
Losing or running out of medications	• Availability of medication stock with the CHW • After consulting the OP, the CHW will replenish the patient’s medications
Stopping medications due to side effects	• Change in medications by the CHW after consulting the OP when there are side effects due to medications
Affordability of medicines	• Free medications

*CHW* Community Health Worker, *OP* Outreach Physician

The intervention will include the following four components:

1. *House-to-house screening for hypertension, diabetes and stroke by community-level workers*

**Fig. 3:**
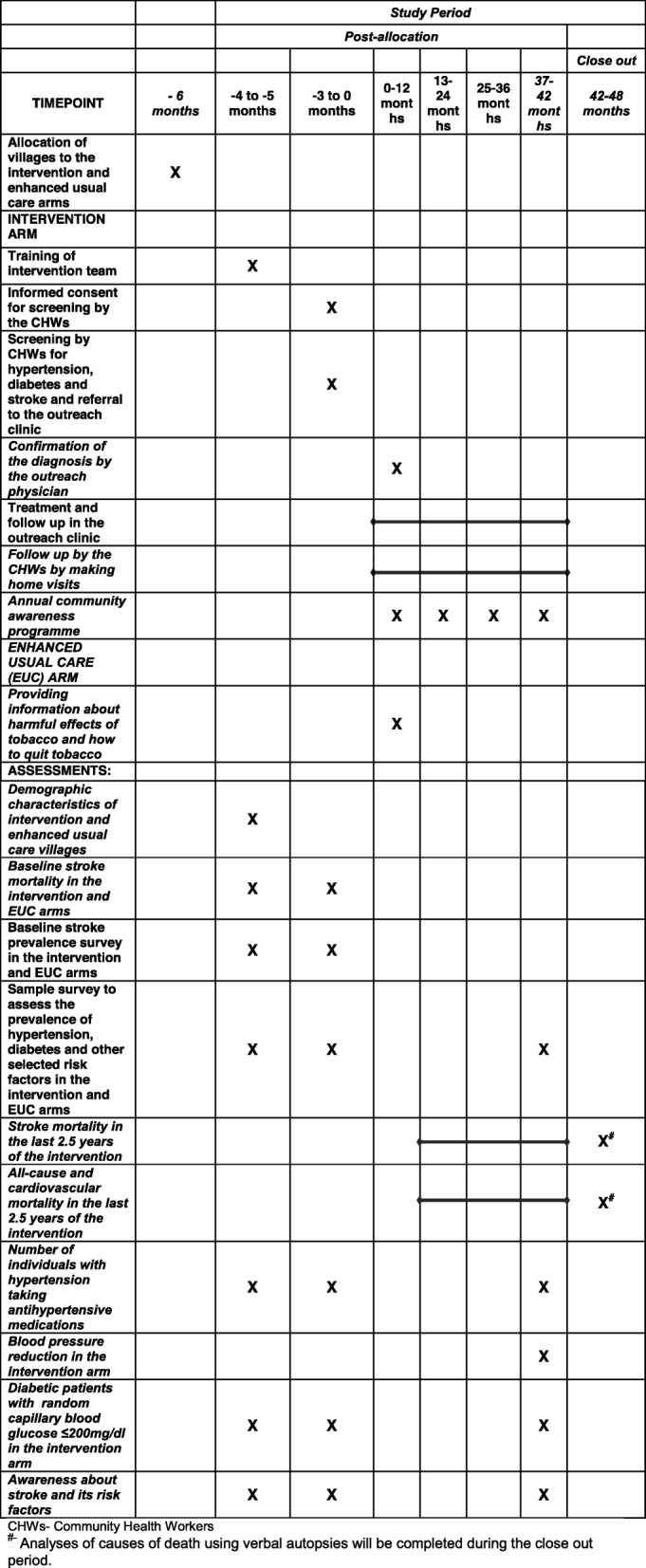
Schedule of enrolment, interventions and assessments (as per Standard Protocol Items: Recommendations for Interventional Trials (SPIRIT) Figure

Female community members, aged 25–40 years and with 7–12 years of school education, will be selected. We plan to select women community health workers for three reasons. First, in this region men often travel outside the villages for small jobs in non-farming seasons and, hence, are not available in the village. Second, one of the responsibilities of the CHW is to counsel patients to quit tobacco and alcohol. In Gadchiroli, tobacco consumption is very common among men and less so in women [34], whereas alcohol use is almost completely restricted to men [5, 6]. Therefore, finding a male worker who will not be using alcohol and tobacco would be very challenging. Third, the acceptance of a male worker examining women is low in this traditional community. We will select one CHW each for villages with a population ≤1500 and two for villages with population >1500. After a rigorous selection process they will be recruited and trained. The CHWs will visit every household with individuals ≥50 years of age and after obtaining informed consent will do the following:

- measure the participant’s blood pressure using an electronic blood pressure monitor (Omron HEM-7121) after the patient has been seated for 5 min. If the initial systolic blood pressure (SBP) is ≥140 mmHg and/or diastolic blood pressure (DBP) is ≥90 mmHg, another reading will be taken at 5 min after the first reading. The average of the two readings will be considered as the blood pressure of the patient.
- check urine glucose on all individuals ≥50 years of age using urine dipsticks
- measure weight, height, waist and hip circumference
- refer patients with hypertension (defined above) or glucosuria (urine glucose more than trace on urine dipstick) to the outreach clinic for evaluation and initiation of treatment
  Stroke patients will be screened using a validated questionnaire as described by us earlier [6]. It had a sensitivity of 85.7% and specificity of 99% in diagnosing stroke in the community where the trial is being conducted [6]. Given the logistics of the trial, the initial house-to-house screening for stroke will be conducted during the baseline assessment of stroke prevalence in the intervention arm (Figs. 2 and 3) by the community-level stroke surveyors, who will be a part of the evaluation team under the trial and will be separate from the intervention CHWs (Additional file 1: Figure S1). This will be done to avoid the inconvenience caused to the target population due to double screening by the community-level surveyors and the CHWs. The community-level surveyors will screen all individuals ≥50 years of age in the intervention villages for symptoms of stroke using a questionnaire which will inquire about whether any of the family members ever had a) weakness on one side of the body, b) numbness on one side of the body, c) drooping of face on one side, or d) slurring of speech. If any of these have occurred, the respondent will be asked d) if these symptoms were acute in onset and e) whether they lasted more than 24 h. Individuals with one or more of the first four symptoms which were acute in onset and lasted >24 h will be suspected of having had a stroke and will be referred to a stroke survey physician for confirmation of the diagnosis.

2. *Evaluation, guideline-based treatment and follow up of patients with hypertension, diabetes and stroke by a mobile outreach clinic and referral where necessary*

A mobile outreach clinic comprising an outreach physician (OP), a pharmacist and a driver will visit each intervention village once every 2 to 3 months. The OP will evaluate patients referred by the CHWs, confirm diagnosis of hypertension by rechecking blood pressure, diagnose diabetes by checking random capillary blood glucose (RCBG) using a glucometer (Accu-Chek Active) and clinically confirm the diagnosis of stroke using WHO’s definition of stroke as previously described [6]. Hypertension will be treated using hydrochlorothiazide, amlodipine and atenolol; diabetes, with metformin and glipizide (Tables 2 and 3). At the beginning of the trial, a stroke survey physician will confirm the diagnosis of stroke, and a list of stroke patients will be provided to the OP to evaluate these patients and start treatment. Stroke patients will receive secondary prophylaxis using low-dose atorvastatin (10 mg) and enteric-coated aspirin (75 mg) as per the treatment algorithm of the study (Tables 2 and 3). Stroke patients who had loss of consciousness or seizures at the onset of symptoms will be presumed to have had a haemorrhagic stroke. These patients and those with intra-cerebral haemorrhage on brain imaging will not be prescribed aspirin. The goal of the anti-hypertensive treatment will be to maintain blood pressure <140/90 mmHg among those with hypertension and <130/80 mmHg among those with diabetes and chronic renal failure but to keep it >100/60 mmHg. Among diabetes patients, the target will be to keep RCBG between 100 and 200 mg/dL. Patients with stroke, if they have hypertension or diabetes, will be treated according to the guidelines for treatment of these conditions. All the trial medicines, urine and blood glucose testing will be provided free of cost to the patients during the study period. The OP will refer patients to the rural hospital of SEARCH if hypertension or diabetes cannot be controlled with trial medications, if patients are unable to tolerate any of the trial medications or when cardiac diseases (angina, atrial fibrillation or heart failure) or renal failure are clinically suspected. If screened individuals or patients who are being followed up develop acute stroke during the intervention period, they will be started on treatment for secondary prevention of stroke once they are back to their homes after receiving acute care for stroke. Also, individuals who did not have hypertension or diabetes at the time of screening but are incidentally diagnosed with hypertension or diabetes at a later date by the OP or another physician during the study period and wish to take treatment from the outreach clinic will be enrolled in the intervention and will receive treatment as per the study treatment algorithm. Participants will be allowed to continue medications for other ailments from their regular physicians. If participants wish to take medications for hypertension, diabetes or stroke from other physicians but wish to get their blood pressure or blood glucose checked by the OP, they will be allowed to do so if they provide a consent. Patients who wish to discontinue the treatment provided in the trial will be free to do so at any point during the trial.

**Table 2:** Formulary of trial medications

Drug	Starting dose	Increments	Maximum dose	Tablet strengths	Dosing frequency per day
Hypertension					
HCT	12.5 mg	12.5 mg	25 mg	12.5, 25 mg	1
Amlodipine	5 mg	2.5, 5 mg	10 mg	2.5, 5 mg	1–2
Atenolol	25 mg	25 mg	50 mg	25, 50 mg	1
Diabetes					
Glipizide	5 mg	2.5 mg	20 mg	5 mg	1–2
Metformin	500 mg	250 to 500 mg	2000 mg	500 mg	1–2
Stroke					
Aspirin	75 mg	–	–	75 mg	1
Atorvastatin	10 mg	–	–	10 mg	1

*HCT* Hydrochlorothiazide

**Table 3:** The preferred sequence of medications by selected criteria

Criterion	First line	Second line	Third line
Hypertension			
Age < 65 years	HCT	Amlodipine	Atenolol
Age ≥ 65 years	Amlodipine	Atenolol	HCT
Diabetes			
BMI < 19	Glipizide	Metformin	–
BMI ≥ 19	Metformin	Glipizide	–
Stroke			
Loss of consciousness or seizures at the onset of stroke or ICH among those who had brain imaging	Atorvastatin	–	–
All other stroke patients	Aspirin Atorvastatin	–	–

*HCT* Hydrochlorothiazide, *ICH* Intra-cerebral haemorrhage

3. *Follow up by the CHW to ensure medication compliance, risk factor control and health education*

The CHW will follow each patient with hypertension, diabetes and stroke once a month to assess if the patient is taking medications as prescribed and tolerating them and whether the blood pressure is adequately controlled (<140/90 mmHg). The CHW will also counsel patients not to have added salt during meals and quit tobacco and alcohol. CHWs will have a stock of the trial medications available with them. If blood pressure control is inadequate or the patient is not tolerating a medication, then the CHWs will inform the OP over phone and make modifications to the treatment as instructed. This will be done to ensure that side effects are addressed in a timely manner, and hypertension control is ensured before the next village visit of the OP. If a patient fails to attend the mobile clinic, then the OP will instruct the CHW to provide medications prescribed during the previous visit to ensure an uninterrupted supply of medications. CHWs will also provide individual health education to patients regarding hypertension, diabetes and stroke using videos and animations in the local Marathi language specifically designed for this purpose. These videos and animations will be shown using hand-held computers. In every home visit, the CHW will inquire about current alcohol and tobacco use among the patients. She will provide information regarding adverse health effects of tobacco and alcohol, counsel the patient to stop these and give tips to quit. Patients with hypertension, diabetes or stroke who do not wish to take their medications from the mobile outreach clinic but consent to follow up by the CHWs will also be visited every month and provided health education and counselling. If screened individuals or patients being followed develop stroke, they will be screened by the CHWs and referred to the OP if they meet the screening criteria for stroke as previously described [6]. The work of the CHWs will be supervised by two field supervisors who will visit every CHW once every 15 days.

4. *A community awareness programme to provide health education about stroke and its risk factors*

The awareness programme will be designed based on the insights gained in the formative study on the knowledge, attitudes and practices for stroke [23]. The information on hypertension, diabetes and stroke will be provided through short movies and animations developed in the local Marathi language for this purpose. The awareness programmes will be conducted by field supervisors once every year in each intervention village. The supervisors will conduct these sessions in the evening after announcing the programme, and it will be open for all villagers to attend.

### Enhanced usual care

The usual care in the villages in this arm includes the availability of free screening and treatment facilities for hypertension and diabetes and secondary prevention of stroke at the PHCs, rural hospitals and district hospitals under the government’s NPCDCS programme. Villagers also seek care at private providers in nearby towns which are the block headquarters or the district sites. In addition to this care, all households in this arm will be provided information pamphlets regarding the harmful effects of and information on how to quit tobacco use at the beginning of the intervention. This will be done because nearly 60% of individuals ≥50 years of age use tobacco in this district [34]. The villages in this arm are also a part of the campaign of SEARCH to reduce tobacco and alcohol use in the entire district, as discussed earlier.

Details on the services available for treatment of hypertension and diabetes, for prevention of stroke and for alcohol and tobacco control in both the arms are as shown in the Additional file 2: Table S1.

### Adverse events

As this is a pragmatic trial, and because we are using standard medications, we anticipate the adverse events commonly reported with these medications. We will use case definitions, standardised operating procedures and a reporting protocol to record all adverse events and treatment of these will be covered under the trial.

### Primary and secondary outcomes and evaluation of outcomes

The primary outcome of the trial will be a reduction in stroke mortality. We will compare stroke mortality in the two arms in the last 2.5 years of the intervention period. We selected stroke mortality as the primary outcome for two reasons: first, the trial was planned in an attempt to address a local public health problem, i.e. high stroke mortality in rural Gadchiroli [5], and second, because non-randomised studies of community-based interventions from Taiwan and Japan have shown the feasibility of reducing stroke mortality over a reasonably short period of time [35, 36]. All deaths will be recorded by the demographic surveillance system. Stroke deaths will be determined using verbal autopsies as previously described [5, 28]. Verbal autopsies will be conducted on all deaths in the intervention and EUC arms during the intervention period using a verbal autopsy tool which was validated in the Million Death Study [37]. Verbal autopsies have relatively high sensitivity (≥75%) and specificity (>90%) in diagnosing stroke in validation studies conducted in India and other countries and remain an important tool to assess stroke mortality in resource-poor settings [38, 39]. Verbal autopsies will be coded by two trained physicians independently. If the two coders do not agree, a third physician will adjudicate the cause of death. Verbal autopsy coders and adjudicators will be blinded to the identity and location of the deceased. For diagnosing death due to stroke, verbal autopsy coders will use the definition of stroke provided by the WHO [30]. Deaths with diagnostic codes of I64 through I69 as per the tenth revision of the International Classification of Diseases, (ICD-10) will be counted as stroke deaths [5]. Stroke deaths in each arm between July 1st 2017 and December 31st 2019 will be considered. Stroke mortality rate per 100,000 population in individuals ≥50 years of age will be estimated and compared.

The secondary outcomes include the following:

- reduction in all-cause and cardiovascular (ICD 10 codes I00-I99) mortality in the intervention area in the last 2.5 years of the intervention compared to that in the EUC arm over the same period. These will be estimated using the method of verbal autopsy as described above.
- percentage of hypertensive patients taking antihypertensive medicines
- blood pressure reduction in the intervention arm
- percentage of diabetic patients who have random capillary blood glucose of 200 mg/dL or less in the intervention arm
- awareness about stroke and its risk factors, determined by the respondents’ answers to two questions. Which body organ is affected in stroke? Which diseases or factors increase the risk of stroke? The appropriate answer to the first question would be brain, and for the second question, any of the following responses, namely hypertension, diabetes, obesity, cardiac ailments, lack of physical activity, tobacco use, alcohol use, consumption of excessive salt, lack of fruits and vegetables in the diet and mental stress, would be correct.

The outcomes b, d and e will be assessed in the intervention and the EUC area at baseline and at the end of 3.5 years of intervention (Fig. 3) through a survey conducted on a randomly selected subsample of individuals ≥50 years of age from both the arms (Fig. 2). The outcome c will be assessed at baseline and at the end of 3.5 years among those receiving treatment in the intervention arm (Fig. 3). The prevalence of risk factors for stroke, including tobacco and alcohol use, blood pressure and glucose control as well as medication adherence and awareness about stroke, will be evaluated in these sample surveys (Fig. 2). In addition, in the sample survey conducted at the end of the intervention, information on any change in medications or side effects due to the medications used for hypertension, diabetes and stroke and any hospitalizations in the past one year will also be collected.

We will estimate prevalence of stroke in two arms at baseline and at the end of the intervention using a three-stage survey as previously described [6]. Briefly, a house-to-house screening for symptoms of stroke will be conducted using a well-validated questionnaire. Diagnosis of stroke will be made by a trained stroke survey physician using WHO’s clinical definition of stroke [30]. Clinical diagnosis alone will be used when no supporting documents are available, irrespective of whether the patient had any residual neuro-deficit at the time of evaluation by the physician. Assessment of disability among stroke survivors will also be done using the modified Rankin Scale. Doubtful cases will be evaluated by an external neurologist. An evaluation of all study outcomes will be conducted by an evaluation team which will be separate from the study implementation team (Additional file 1: Figure S1).

### Strategies to reduce contamination

The likelihood of contamination will be low as villages are the units of randomization and are physically separated. Also, the intervention will be limited to the residents of the intervention villages. The list of the eligible village residents will be provided to the CHWs as well as to the OP based on the population register maintained by SEARCH. The CHWs and the OP will be actively instructed to provide medications only to those who belong to the intervention villages.

### Impact evaluation

#### Sample size

We calculated the sample size based on the primary outcome of the trial, which is a reduction in stroke mortality. We hypothesized that the community-level intervention will produce at least a 40% reduction in stroke mortality over the last 2.5 years of the intervention. This assumption was based on a study in Taiwan where a community-based hypertension control programme resulted in a 40% decrease in stroke mortality rate in the entire population over 3 years [36]. As we plan to measure stroke mortality in only the high-risk population (≥50 years of age) with relatively less access to care and because the intervention uses strategies for both primary and secondary prevention, we assume that the intervention will result in at least 40% reduction in stroke mortality in the given period. We used the formula by Hayes and Bennett to estimate the sample size [40]. We assumed the between-cluster correlation coefficient of variation (k) for stroke mortality to be 0.2, as such data were not available from population-based studies. The baseline crude stroke mortality rate in individuals ≥ 50 years of age was taken as 688/100,000 population based on our previous study in these villages [5]. To detect a 40% reduction in baseline stroke mortality in a population ≥50 years of age with 80% power, at a 5% level of significance and with a *k* of 0.2, we needed 27 villages per intervention group. After adjusting the sample size for 75% coverage, the required sample size was 32 villages per group.

The prevalence of the risk factors for stroke, awareness about stroke and hypertension and blood glucose control in both the arms will be evaluated in sample surveys on a randomly selected subsample of individuals ≥50 years of age. The sample will be drawn from the list of individuals in this age group available in the census conducted by SEARCH in 2015. We calculated the sample size for this survey while assuming that 40% of the individuals aged ≥50 years will have hypertension, and among these, at the end of the intervention, 65% and 35% of the hypertensive individuals in the intervention and EUC arm, respectively, will have their blood pressure controlled (< 140/90 mmHg). Using 80% power, a 5% level of significance, a design effect of 1.6 and a non-response of 20%, the required sample size was 330 individuals per arm.

### Data collection and management

Information on individuals ≥50 years of age will be obtained from the census conducted by SEARCH. Information on stroke deaths will be collected using verbal autopsies, and stroke prevalence will be estimated using a house-to-house survey as described above. Quantitative data will be collected in the field by the CHWs, field supervisors, OP and surveyors of the sample surveys using standardized pre-tested questionnaires. This will be done to assess the indicators specified in Fig. 3. We will monitor the progress of the study and response of the community to each component of the intervention using process indicators which will be captured through standardised management information system (MIS) reports. These indicators will provide key information regarding the operational aspects of the study which can be used for mid-course corrections and for scaling up of the intervention if the intervention is effective. Qualitative data will be collected to explore the reasons for success or failure of the various components of the intervention package. Four to six focus group discussions (FGDs) will be conducted with participants and their relatives, and individual interviews will be conducted with the CHWs, field supervisors and OP to understand the implementation process and facilitators and barriers to the implementation of the intervention. After the paper forms have been checked and the data entered, the forms will be kept in locked cupboards. Data will be stored in databases on a central server in the research department of SEARCH and will be backed up at regular intervals. Qualitative data will be audio recorded, and the audio files will be kept on a computer which is password protected. Access to intervention information will be limited to the study team. Databases will only be accessible to the data management team of the study. We will make the research data from this study available for use by other researchers after publication of trial results in academic journals, and this information will be publicised through research manuscripts.

### Data analysis

The analysis and presentation of the results will be according to the Consolidated Standards of Reporting Trials (CONSORT) Statement for cluster randomised controlled trials [41]. We will first compare baseline demographic features in the intervention and EUC villages such as total population, population ≥50 years of age, sex distribution in this population, socioeconomic and education status and the average distance from the district town to assess whether any baseline imbalances exist after randomisation. We will also compare stroke mortality; point prevalence of stroke; and the prevalence of selected risk factors for stroke, such as hypertension, diabetes, use of tobacco and alcohol, body mass index, waist–hip ratio and awareness about stroke in both the arms.

The impact of the intervention in the intervention villages will be analysed by comparing the primary and secondary outcomes in these two arms under the intention-to-treat principle with use of two-sided tests and a significance level of 5%. Cluster level, as well as individual level data, will be used to compare primary and secondary outcomes. Results will be presented as effect sizes (difference in means between arms, odds ratios). Unadjusted and adjusted results will be presented. We will use regression methods applying generalized estimation equation or logistic regression with random effects models to account for the clustered design of the intervention. Age, sex, occupation, socioeconomic status, education, and baseline stroke mortality will be used as covariates in the multivariate analyses based on their bivariate association with the outcomes of interest. We will also use the Cox proportional hazards regression to compare time to first occurrence of death due to stroke and cardiovascular diseases, as well as any-cause death between the two groups. Pre-planned subgroup analyses will include assessing the effect of the intervention by age, sex, socioeconomic status, education, individual risk factors (hypertension, diabetes, tobacco use, alcohol use, waist hip ratio, and BMI), antihypertensive medication use, number of visits to the outreach clinic and number of visits by the CHW. These will be interpreted with caution. In addition, we will also evaluate the effect of the intervention on disability due to stroke among stroke survivors in both the arms. Pre-planned exploratory analysis will include a Bayesian analysis of the primary trial outcome [42]. Given that we will be working closely with the community, we expect that the amount of missing data will not be large, and hence, we will not have to account for it in analyses. Data will be analysed using statistical software Stata Version 14 (College Station, TX, USA).

Qualitative data will be transcribed and analysed using a thematic approach as described earlier [23].

### Economic evaluation

All intervention-related costs will be audited through the project accounting system. We will estimate the cost per stroke, cardiovascular and all-cause death averted and the cost of delivering these interventions per 100,000 population per year.

### Process evaluation

Process evaluation will be conducted to understand a) how the intervention was delivered, b) the barriers and facilitators for the implementation of the intervention and c) the mechanisms through which the intervention may or may not have worked. Quantitative data from process indicators, review of various process documents and qualitative data obtained through FGDs and individual interviews with participants, their relatives, and the implementation team members will be used for this purpose.

### Quality control measures

Quality control measures will be taken at all levels of intervention delivery, data collection and entry. These include a) rigorous training and evaluation of the CHWs, field supervisors and the OP; b) use of pre-tested standardized questionnaires to collect data by the surveyors, the CHWs and the OP; c) refresher training of the implementation team members at periodic intervals; d) crosschecking as well as re-survey of 5% of the screened population during screening, as well as follow up visits by the CHWs, to check for the accuracy of data collection; e) evaluation of adherence to treatment protocol by the OP; f) range check on the data values; and g) cross-checking of entered data. Blood pressure monitors, glucometers and weighing scales will be calibrated every 1 to 3 months, and equipment showing errors as per pre-defined criteria will be replaced.

### Interim analyses or stopping rules

As we will be using standard medications in the intervention, we have not planned for any interim analyses. However, we will conduct an urgent safety review of the study if, in the intervention arm, serious adverse events are reported by >5% or if minor side effect are reported by >25% of the study participants or if the crude death rate in the intervention arm exceed one standard deviation above the baseline crude death rate (average of crude death rates over 3 years before the intervention) in the intervention villages.

### Trial management

The implementation of the trial will be overseen by the trial execution committee and a trial steering committee. Data and safety will be monitored by an independent external data safety and monitoring board (DSMB), which will review the trial and adverse events data every 6 months.

### Dissemination of findings

We will communicate the results of the trial to study participants, district health officials, state and national health ministries, the national chronic diseases programmes and the Indian Council of Medical Research. The study findings will be presented in national, regional and international conferences. We plan to publish the results of the trial in a PubMed-listed peer-reviewed journal in an open access format.

## Discussion

Stroke is the second leading cause of death globally and has emerged as an important public health priority in rural parts of India, where healthcare services are inadequate [5, 7, 43]. In these resource-poor areas, community-based interventions involving community members could be an important strategy to reduce stroke mortality. Such interventions have been shown to be effective in reducing infant mortality and controlling infections [44–47]. However, a paucity of evidence exists regarding the effectiveness of community-based interventions for chronic non-communicable diseases in rural areas of LMICs [48]. Hypertension is one of the key risk factors for stroke, and recent trials show that community-based approaches for hypertension control could be useful [49–51]. The primary outcomes of these trials have been a reduction in blood pressure and not a reduction in cardiovascular events. To our knowledge this is the first cluster randomised controlled trial from a rural area of a LMIC to evaluate the effect of such an intervention to reduce stroke mortality. Our study thus provides an important value addition over previous studies by combining primary and secondary prevention of stroke at the community level and going a step further to evaluate if such an intervention would reduce stroke mortality in a resource poor setting. The insights gained from this study will provide important inputs for the Government of India’s ambitious programme for the prevention and control of chronic diseases (NPCDCS), which is one of the largest programmes in the world of its kind to reduce the risk of non-communicable diseases. This programme currently focuses on community-level screening and facility-level treatment of chronic diseases but currently does not have a component of community-level follow up. Our study will help to evaluate the importance of community-level treatment and follow up. If successful, the study intervention can be scaled up in other rural and under-resourced settings in the world.

### Trial status

The trial will be recruiting patients until December 2019. The final outcome assessment of all outcomes will be completed by the end of June 2020. Protocol version 1.2; 15 January 2016.

## Supplementary information

**Additional file 1: Figure S1.** Trial manpower and management.

**Additional file 2: Table S1.** Interventions and availability of services in the intervention and the enhanced usual care arms.

**Additional file 3.** SPIRIT 2013 Checklist: Recommended items to address in a clinical trial protocol and related documents*.

## Data Availability

All data underlying the results are available as part of the article, and no additional source data are required.
