# Supplementary material for: Effect of a community-based intervention for cardiovascular risk factor control on stroke mortality in rural Gadchiroli, India: study protocol for a cluster randomised controlled trial
Source: Trials. 2019 Dec 23;20:764. doi: 10.1186/s13063-019-3870-x (PMC6929484; doi:10.1186/s13063-019-3870-x)

Supplementary Figure 1

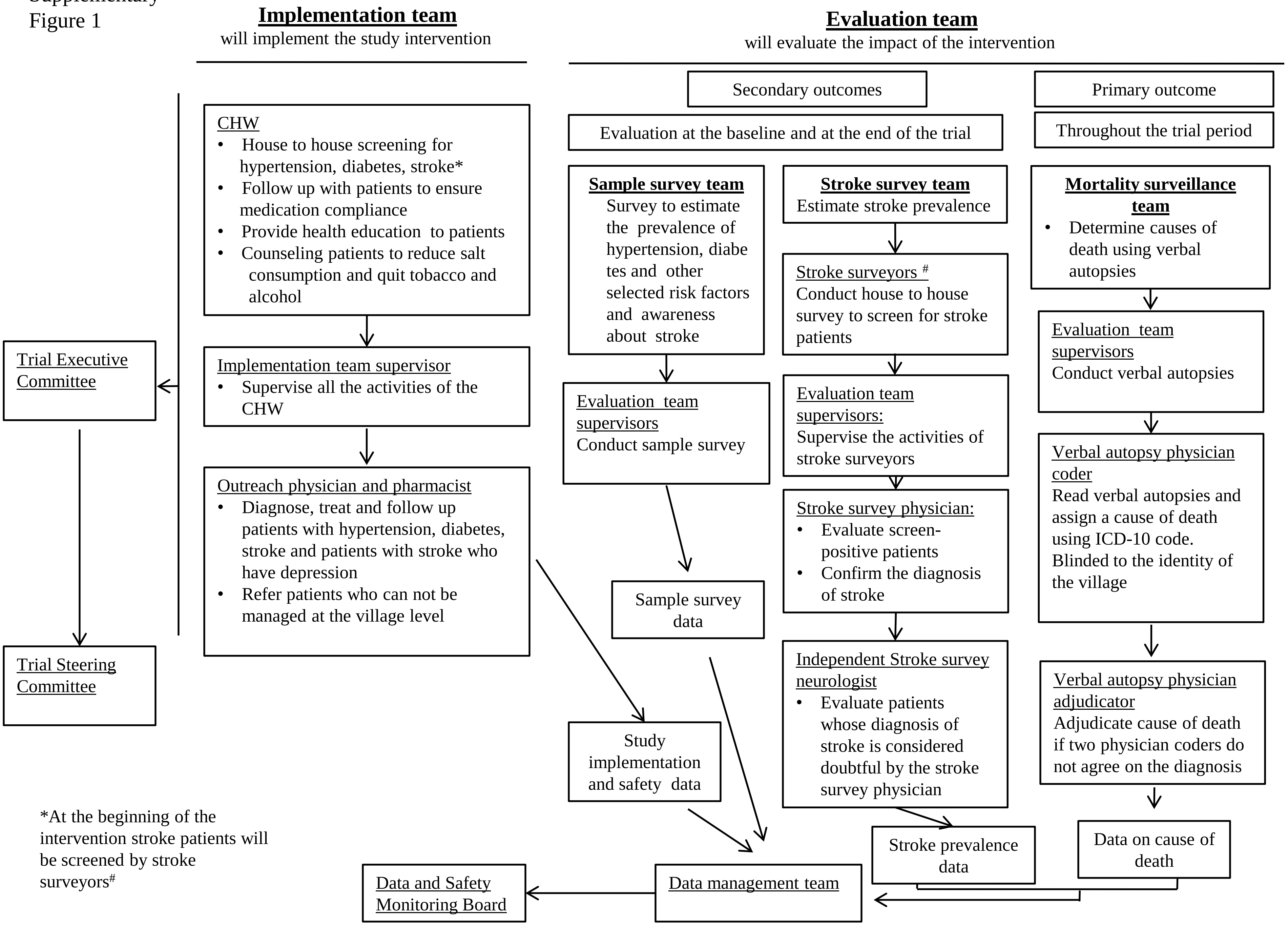

Supplement: Supplementary file 1 — Additional file 1: Figure S1. Trial manpower and management. [file 13063_2019_3870_MOESM1_ESM.pdf]
