# Supplementary material for: Effect of a community-based intervention for cardiovascular risk factor control on stroke mortality in rural Gadchiroli, India: study protocol for a cluster randomised controlled trial
Source: Trials. 2019 Dec 23;20:764. doi: 10.1186/s13063-019-3870-x (PMC6929484; doi:10.1186/s13063-019-3870-x)
Supplement: Supplementary file 2 — Additional file 2: Table S1. Interventions and availability of services in the intervention and the enhanced usual care arms. [file 13063_2019_3870_MOESM2_ESM.docx]

**Additional file 2: Table S1. Interventions and availability of services in the intervention and the enhanced usual care arms**

|  | **Intervention** | **Enhanced usual care** |
| --- | --- | --- |
| Screening for hypertension and diabetes | - Active house to house screening by community health workers (CHWs) - Facilities available free of cost at the primary health centres, rural and district hospitals of the government health system - Private practitioners | - Facilities available free of cost at the primary health centres, rural and district hospitals of the government health system - Private practitioners |
| Treatment for hypertension, diabetes and secondary prevention of stroke | - Free treatment through the mobile village clinic under the intervention - Free treatment available at the primary health centres, rural and district hospitals of the government health system - Private practitioners | - Free treatment available at the primary health centres, rural and district hospitals of the government health system - Private practitioners |
| Follow up for hypertension, diabetes and stroke care | - Home visits by CHWs - Free follow up care at the primary health centres, rural and district hospitals of the government health system - Private practitioners | - Free follow up care at the primary health centres, rural and district hospitals of the government health system - Private practitioners |
| Tobacco and alcohol cessation | - Individual counselling by the CHWs regarding adverse effects of tobacco and information on steps to quit - Mass awareness generation, reducing supply through administrative actions, community mobilisation and de-addiction services at the rural hospital of SEARCH under a district wide alcohol and tobacco cessation campaign of SEARCH | - Information provided to individuals ≥50 years of age regarding adverse effects of tobacco and alcohol and information about steps to quit in each household using information pamphlets at the beginning of the intervention - Mass awareness generation, reducing supply through administrative actions, community mobilisation and de-addiction services at the rural hospital of SEARCH under a district wide alcohol and tobacco cessation campaign of SEARCH |
| Awareness generation about hypertension, diabetes and stroke | - Active awareness generation through individual counselling of patients - Annual community awareness campaign under the intervention - Awareness material available through the national non-communicable diseases programme | - Awareness material available through the national non-communicable diseases programme |
